# Genomic and functional characterization of a *Butyricicoccus porcorum* strain isolated from human gut microbiota

**DOI:** 10.1128/msystems.00790-25

**Published:** 2025-07-09

**Authors:** Ni Zhao, Peiling Geng, Alejandro Gaher Perez, Anakha Chandramana Maya, Brijesh Yadav, Yuxuan Du, Yong Ge

**Affiliations:** 1 Department of Microbiology, Immunology & Molecular Genetics, University of Texas Health San Antonio, San Antonio, Texas, USA; 2 Department of Electrical and Computer Engineering, University of Texas at San Antonio 551331, San Antonio, Texas, USA; University of California, San Francisco, San Francisco, California, USA

**Keywords:** *Butyricicoccus porcorum*, gut microbiome, colonic epithelial cell, cholesterol biosynthesis, T helper 17 cell

## Abstract

The gut is the most complex microbial ecosystem in the body that greatly influences human immune and metabolic health. However, the functional understanding of gut microbiome is hampered by our limited ability to obtain bacterial cultures for experimental validation, particularly low-abundant species that may carry specific functions but are often overlooked by population-based analyses. Here, we isolated and characterized a novel strain of *Butyricicoccus porcorum* (named Bp 531D) from human gut microbiota, representing the first butyrate-producing human isolate within a phylogenetic clade of *Butyricicoccus* complex. Comparative whole-genome analysis revealed a unique capability of Bp 531D for one-carbon metabolism and a high abundance of mobile genetic elements, including six prophages and plentiful transposons, reflecting its evolutionary flexibility. Oral administration of the bacterium profoundly altered gut microbiome composition in C57BL/6 mice, leading to controlled microbial oxidative signaling and calibrated carbohydrate metabolic function in the gut. RNA sequencing (RNA-seq) analysis demonstrated notable functional programming of colonic ECs, whereupon Bp 531D primarily restricted the biosynthesis of cholesterols and activated the pathway of antigen processing and presentation. Furthermore, the expression of MHC class II was correlatively heightened in colonic dendritic cells (DCs), and the frequencies of interleukin-10- (IL-10) and IL-22-producing T helper 17 (Th17) cells were significantly elevated in mice treated with Bp 531D compared to controls. Our findings uncover the crucial roles of *B. porcorum* in supporting intestinal homeostasis and provide a novel functional modulator to potentially optimize microbial strategies for improving intestinal health.

**IMPORTANCE:**

Reduced abundance of the *Butyricicoccus* genus has been associated with human intestinal disorders, including inflammatory bowel diseases. While supplementation of *B. pullicaecorum* mitigates intestinal inflammation, it is unclear whether other *Butyricicoccus* species critically contribute to intestinal microbial and immune homeostasis. We identified a novel *Butyricicoccus* species within human gut microbiota and characterized its detailed intestinal functions using the C57BL/6 mouse model. Our findings may further highlight the genetic and functional diversities of the gut microbiome.

## INTRODUCTION

The human gastrointestinal tract is host to a diverse and dynamic microbial community that influences human physiology and health (1–3). This complex ecosystem is dominated by hundreds of symbiotic bacterial species, most of which are unculturable and thus yet to be characterized (4, 5). Understanding the functions of the gut microbiome, however, requires cultivated bacteria for experimental validation using animal models, thus posing a challenge for mining specific bacteria to be developed for targeted and perhaps personalized probiotics (6). In the human gut, bacterial members of Clostridial clusters IV and XIVa have gained considerable attention as next-generation probiotics largely due to their production of butyrate (7–9). Butyrate is produced through the reactions of bacterial carbohydrate-active enzymes (CAZymes) and plays a crucial role in host metabolic health (10). It not only serves as an energy source for colonocytes (11) but also induces the differentiation of colonic regulatory T cells (Tregs), critically contributing to host immune tolerance (12). However, emerging data also suggest that the *in vivo* functional properties of butyrate-producing microbes may not be confined to or dependent on their butyrate-producing capability (13–15), but heavily rely on host intestinal microenvironments and bacterial genomic variations. This highlights the necessity to obtain and characterize even genetically closely related strains that may exhibit functional specification.

The genus of *Butyricicoccus* comprises four described species (*B. pullicaecorum, B. faecihominis, B. intestinisimiae*, and *B. porcorum*), among which *B. pullicaecorum* is initially isolated from cecal contents of broiler chickens (16) and later found in the human gut microbiome (17). *B. pullicaecorum* is a member of Clostridial cluster IV, and thus far, few animal studies have been conducted solely with this species (18–23). For instance, patients with inflammatory bowel diseases have reduced counts of *Butyricicoccus* in their feces when compared to healthy controls (~10^9^ 16S rRNA copies/g feces), and supplementation of *B. pullicaecorum* decreases lesion sizes and inflammation in a rat model of chemically induced colitis (23). Oral administration of *B. pullicaecorum* provides colonization resistance against pathogens (22) and augments anti-tumor activities (20, 21). Furthermore, a recent human interventional trial with encapsulated *B. pullicaecorum* strain 25-3^T^ (Bpu 25-3^T^) demonstrates its safety and tolerance by healthy volunteers (7), indicating its potential as a next-generation probiotic.

*B. porcorum* is a Gram-positive, non-motile, butyrate-producing coccus first isolated from the distal ileum of a pig in the United States (24). Since then, the bacterium has been repeatedly found in the intestinal tracts of pigs, chickens, and rats (25–27). A recent metagenomic study also reports the *B. porcorum* DNA in the gut microbiome of a Chinese cohort after oral ingestion of a *Bifidobacterium* strain (28). Furthermore, decreased abundances of the *Butyricicoccus* genus (no species-level classification) are associated with several human inflammatory disorders (23, 29). However, it remains unclear how *Butyricicoccus*, including *B. porcorum*, may regulate intestinal cells to resist pathogenic inflammation. In this study, we isolated a novel human *B. porcorum* strain and demonstrated its regulatory functions on gut microbiome, epithelial cells (ECs), and immune response. Our findings thus identify a potential probiotic species with a unique functional capacity to control epithelial cholesterol biosynthesis and immune activation.

## RESULTS

### Isolation and characterization of Bp 531D

**Fig 1:**
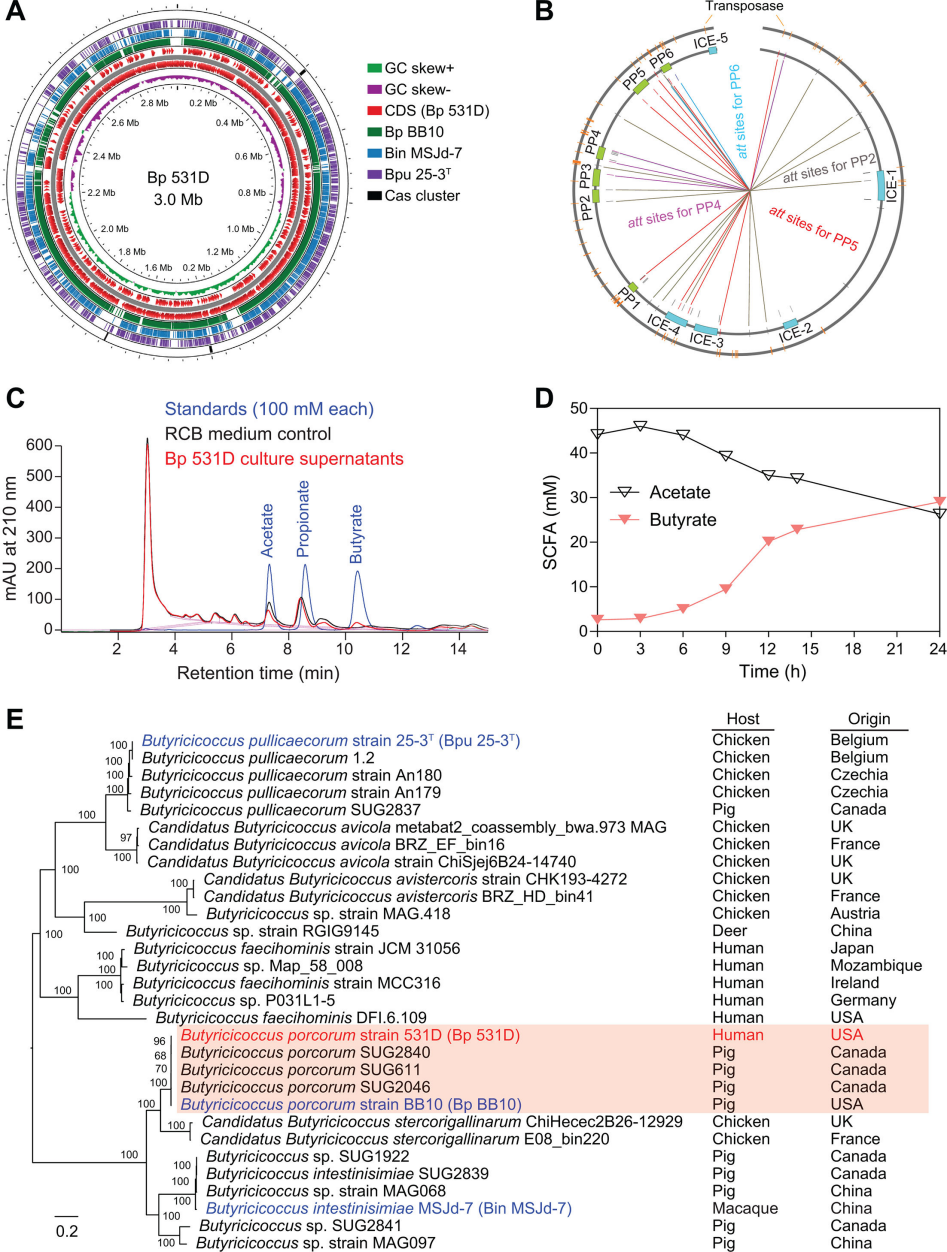
Characterization of Bp 531D isolated from human gut microbiota. (**A**) Genome comparison of *B. porcorum* 531D (Bp 531D) with *B. porcorum* BB10 (Bp BB10), *B. pullicaecorum* 25-3T (Bpu 25-3^T^), and *B. intestinisimiae* MSJd-7 (Bin MSJd-7). Rings with different colors indicate different bacterial genomes. Cas clusters are shown in the outermost ring. (**B**) Overview of mobile genetic elements. Predicted transposases, prophages (PPs), and integrative/conjugative elements (ICEs) are shown on the circles. The attachment (*att*) sites of PPs are indicated with different colors. (**C**) Chromatograms of acetate and butyrate peaks in the Bp 531D culture supernatants compared to the standards and medium control. (**D**) HPLC measurement of acetate and butyrate in the supernatants of bacterial cultures (*n* = 3 samples/time point). (**E**) Whole-genome phylogenetic analysis of representative strains from the *Butyricicoccus* genus. The host origin and country of isolation are shown. The tree was built on the protein and gene sequences for 100 single-copy genes found in selected genomes. Branch support values are obtained using 100 bootstraps, and the length of a branch is relative to the total number of changes at each site. Bacteria highlighted in colors are selected for comparative whole-genome analyses.

Given the pivotal role of butyrate and that strain-specific function often exists (30, 31), efforts were made to isolate novel bacterial strains within butyrate-producing Clostridial clusters. Using strict anaerobic culturing and selective reinforced clostridial medium, a new *B. porcorum* strain (Bp 531D) was isolated from the stools of a healthy human donor. *De novo* assembly using complementary Oxford nanopore long-reads and high-quality Illumina short-reads yielded a single circular genome (2,990,647 bp) (Fig. 1A), representing the first complete genome within the *Butyricicoccus* genus. The genome carries a plethora of transposases, five integrative conjugative elements (ICEs), and six prophages (Fig. 1B), which may recognize the same or different attachment (*att*) sites and drive multiple recombination and transposition events during the evolution process. The crossover of these *att* sites may suggest the existence of various excision and integration processes that might produce different prophage variants under specific environmental pressures (Fig. 1B; Fig. S1). HPLC analysis demonstrated Bp 531D synthesizes butyrate, as one of the major end-products detected in the culture supernatants (Fig. 1C), which was accompanied by the proportional consumption of acetate (Fig. 1D). Whole-genome phylogenetic analysis showed that Bp 531D is classified into a clade wherein most strains were isolated from swine of different countries, whereas *B. pullicaecorum* occupies a separate clade with reported human isolates (Fig. 1E).

### Genomic signatures of *B. porcorum*

**Fig 2:**
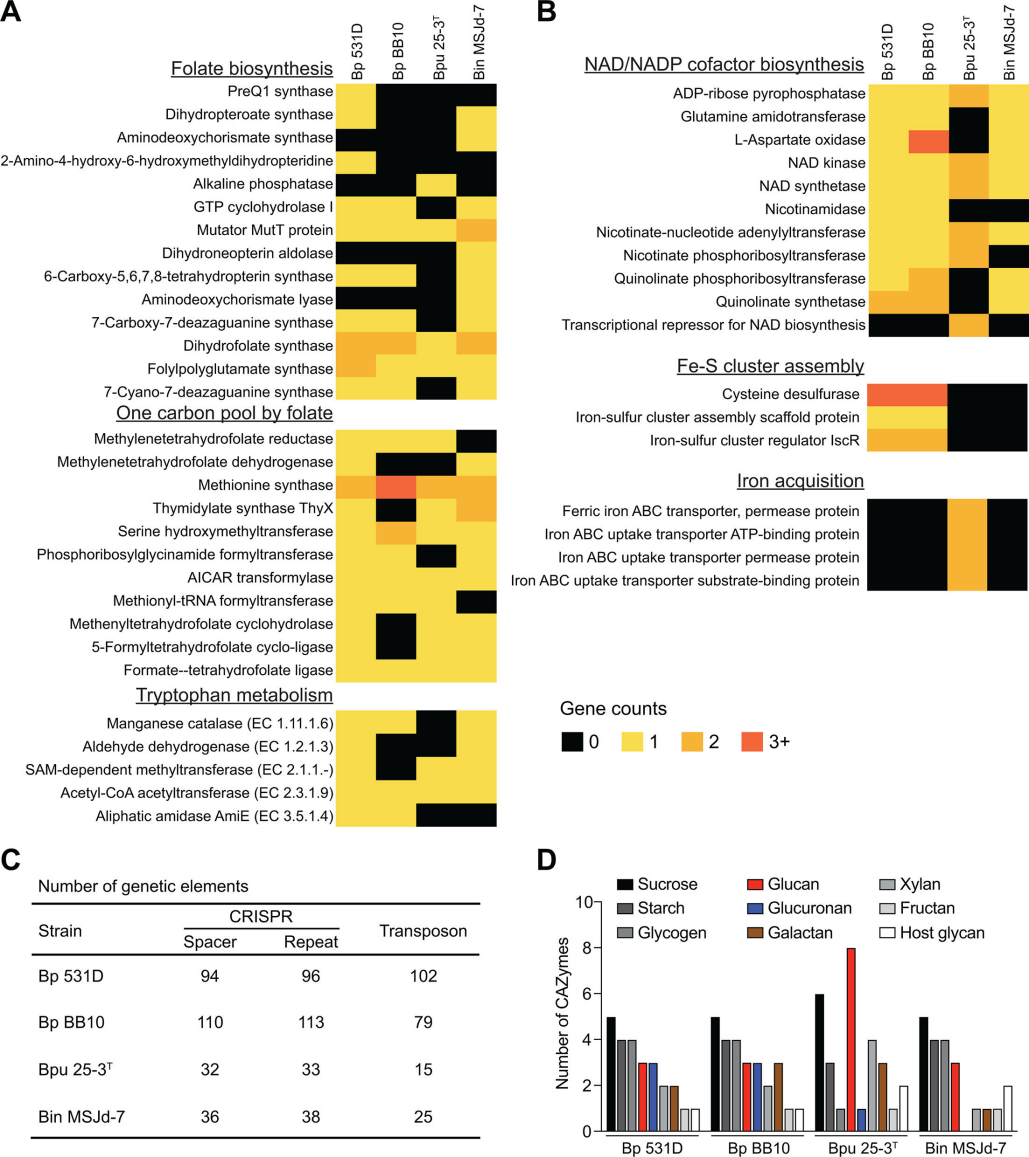
Comparative genome analysis of *Butyricicoccus*. (**A**) Heatmaps of metabolic enzymes of indicated pathways on the genomes of Bp 531D, Bp BB10, Bpu 25-3^T^, and Bin MSJd-7. (**B**) Heatmaps of genes related to the indicated subsystems. (**C**) Summary table of the number of CRISPR spacers/repeats and transposons in the compared genomes. (**D**) The abundance of CAZymes predicted for utilizing indicated carbohydrates.

Next, we selected the genome assemblies of *B. porcorum* strain BB10 (Bp BB10)*, B. pullicaecorum* strain 25-3^T^ (Bpu 25-3^T^), and *B. intestinisimiae* strain MSJd-7 (Bin MSJd-7) and compared them with Bp 531D to identify potential species- and strain-specific genetic signatures. These assemblies have relatively high completeness and quality (scaffold L50 <5 and contig N50 >170 kb) and are therefore likely to carry complete gene information. By comparison of metabolic pathways in different strains, we found that Bp 531D and Bp BB10 differ in folate biosynthesis (Fig. 2A). Specifically, 2-amino-4-hydroxy-6-hydroxymethyldihydropteridine (EC 2.7.6.3) and dihydropteroate synthase (EC 2.5.1.15), involved in sequential conversion of 2-amino-4-hydroxy-6-hydroxymethyl-7,8-dihydropteridine to 7,8-dihydropteroate, a precursor for 7,8-dihydrofolate that enters one-carbon metabolism, are both present in Bp 531D but not in other selected strains. Accordingly, Bp 531D is enriched with enzymes for the one-carbon pool by folate, critical for DNA biosynthesis and methylation reactions. In addition, two tryptophan catabolic genes (aldehyde dehydrogenase and aliphatic amidase) co-exist in the genome of Bp 531D (Fig. 2A).

The functional differences of the genomes were further examined using bacterial subsystems, which are expertly curated sets of biologically related functional roles. Compared to Bpu 25-3^T^ and Bin MSJd-7, the two *B. porcorum* strains have increased genomic capacity for NAD and NADP cofactor biosynthesis and Fe-S cluster assembly, whereas Bpu 25-3^T^ is uniquely equipped with transporters for iron uptake (Fig. 2B). Furthermore, *B. porcorum* harbors high numbers of CRISPR spacers and repeats and carries plenty of transposons (Fig. 2C). The number of transposases and recombination potential were also higher in *B. porcorum* when compared to *B. pullicaecorum* or *B. intestinisimiae* (Fig. 1B; Fig. S2A through C)*,* indicating species-specific evolution and adaptation. Moreover, the distribution of the MGE attachment sites is much more confined in the genome of Bp BB10 compared to that of Bp 531D (Fig. 1B; Fig. S2B). Although there is high convergence and similarity between the two prophages detected in the two *B. porcorum* strains, human-derived prophage (PP4) appears to be a hybrid of 531D-PP2 and 531D-PP3 (Fig. S2D), further highlighting the active recombination dynamics within Bp 531D. In addition, both *B. porcorum* strains were predicted to possess a similar carbohydrate-utilizing profile, with less glucan-degrading but more glucuronan-degrading CAZymes than Bpu 25-3^T^ or Bin MSJd-7 (Fig. 2D), illuminating species-specific metabolic signatures.

### Regulation of gut microbiome composition and function

**Fig 3:**
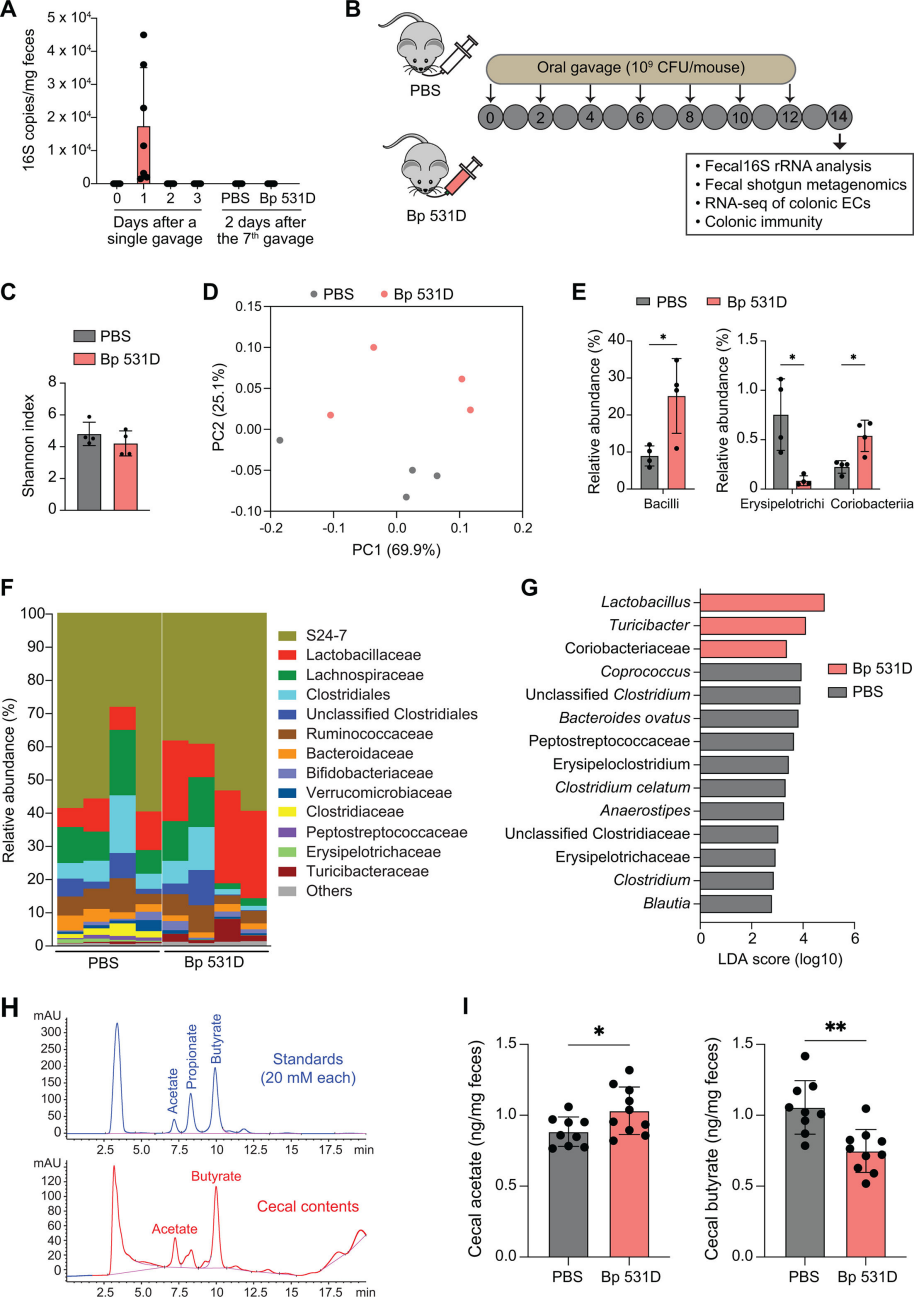
16S rRNA sequencing of fecal microbiome. (**A**) qPCR quantification of Bp 531D-specific 16S rRNA copies in the feces of mice (*n* = 7) after a single oral gavage (10^9^ CFU/mouse), or 14 days after 7 gavages (*n* = 8/group). (**B**) Experimental scheme. Groups of C57BL/6 mice were orally gavaged with Bp 531D (10^9^ CFU/mouse) or PBS every 2 days. Feces were collected from the two groups of mice on day 14 for 16S rRNA and metagenomics sequencing. The transcriptomes of colonic ECs and colonic immune response were also analyzed on day 14. (**C**) Shannon index derived from 16S rRNA analysis of the two groups of fecal microbiotas (*n* = 4/group). (**D**) The weighted UniFrac principal coordinate analysis (PCoA) plot depicts the bacterial community structures. (**E**) Top bacterial class abundance. *P < 0.05, unpaired Student *t*-test. (**F**) The relative abundance of top bacterial families. (**G**) LEfSe plot showing differentiating taxa (species level; LDA score >2) between Bp 531D- and PBS-gavaged mice. (**H**) HPLC chromatograms of acetate and butyrate peaks in the extracts of the standards (top) and cecum contents (bottom). (**I**) HPLC quantification of acetate and butyrate in the cecal contents of mice gavaged with Bp 531D or PBS (*n* = 9-10/group). Data are presented as mean ± SD. *P < 0.05, **P < 0.01, unpaired Student *t*-test.

To elucidate the potential *in vivo* function of Bp 531D, we first tracked the colonization of the bacterium in conventional C57BL/6 mice via oral gavage. No Bp 531D-specific 16S rRNA was detected 2 days after a single gavage or 14 days after 7 gavages (Fig. 3A), documenting a transient gut colonization. Thus, we treated mice with Bp 531D or PBS every 2 days for 14 days to profile the gut microbiome, colonic ECs, and immune responses (Fig. 3B). 16S rRNA sequencing showed that oral administration of Bp 531D had no impact on α-diversity (Fig. 3C), but led to a shift in the overall microbial community (Fig. 3D). The relative abundance of the class Bacilli was increased from 8.9% in the PBS group to 25.1% in the Bp 531D group, and differential abundances of Erysipelotrichi and Coriobacteriia were also observed (Fig. 3E). At the family level, Lactobacillaceae was significantly elevated, and Clostridiaceae was almost depleted by the bacterial gavage (Fig. 3F). LEfSe analysis demonstrated the enrichment of *Lactobacillus* and *Turicibacter*, while *Bacteroides ovatus* and several *Clostridium* taxa (e.g., *Clostridium celatum*) were reduced in Bp 531D-gavaged mice compared to PBS controls (Fig. 3G). Knowing that members of the Clostridiaceae family produce butyrate in the gastrointestinal tract (32, 33), we next quantified the level of butyrate in the cecal contents of mice gavaged with Bp 531D versus PBS (Fig. 3H and I). Here, the depletion of Clostridiaceae was correlated with a significant reduction of cecal butyrate and a concurrent accumulation of acetate (Fig. 3I), illustrating a functional impact on the microbiome.

**Fig 4:**
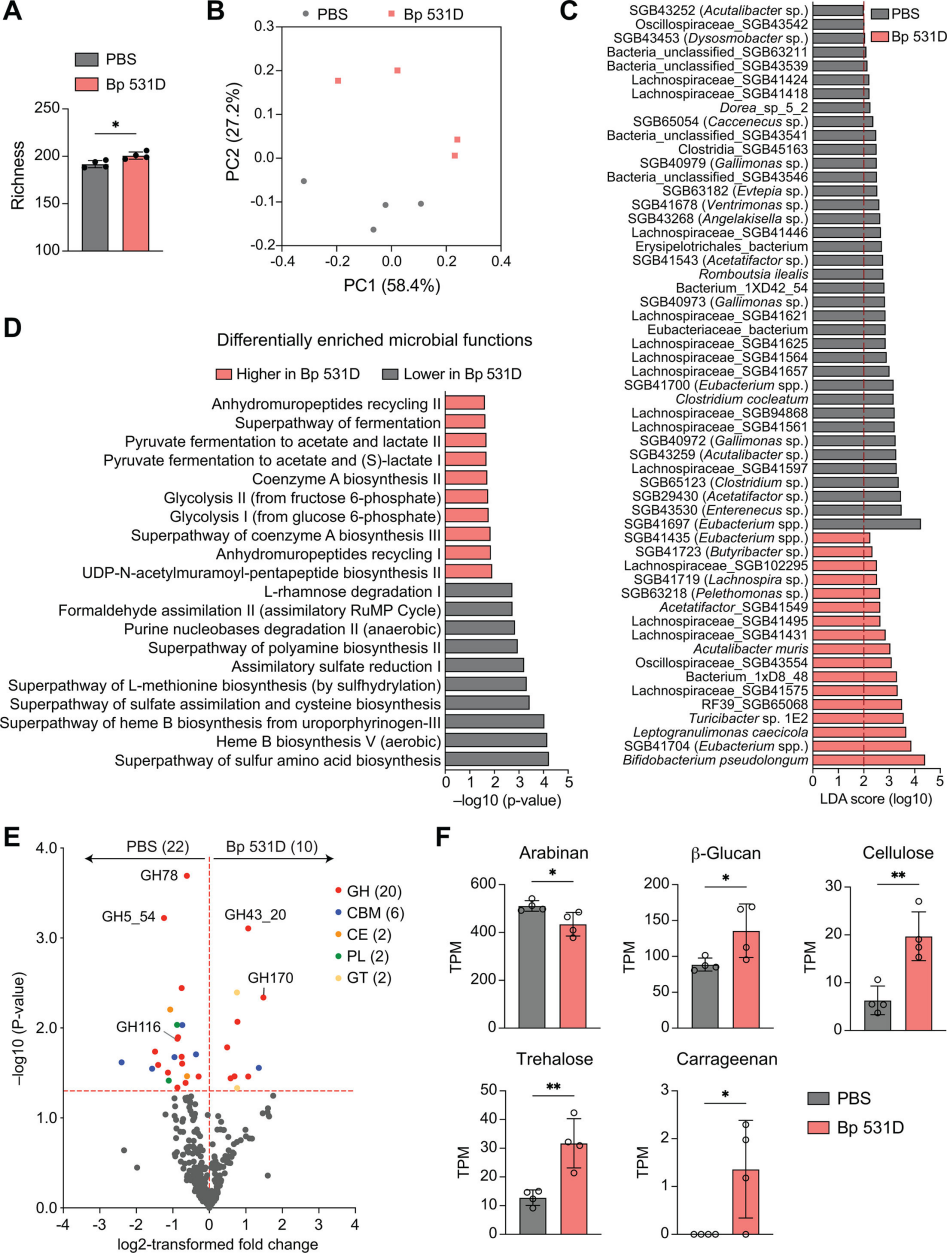
Functional metagenomic profiling of fecal microbiota in mice treated with Bp 531D. (**A**) Bacterial richness in the fecal metagenomes of Bp 531D- and PBS-gavaged mice (*n* = 4/group). (**B**) PCoA plot based on Bray-Curtis distances between the two groups of metagenomes. (**C**) LEfSe plot showing differentiating taxa at the species level. Annotations using the Genome Taxonomy Database (GTDB) are included in parentheses. SGB, species-level genome bin. (**D**) The abundances of bacterial functional pathways are differentially enriched in Bp 531D-gavaged mice compared to PBS controls. (**E**) Volcano plot of CAZymes detected in the metagenomes. The horizontal dashed red line denotes the *P*-value of 0.05. GH, glycoside hydrolase; CBM, carbohydrate-binding module; CE, carbohydrate esterase; PL, polysaccharide lyase; GT, glycosyltransferase. (**F**) The total abundance of CGCs predicted to utilize the indicated substrates. TPM (Transcripts Per Million) is used as a measurement of normalized sequence reads mapped to CAZymes. *P < 0.05 and **P < 0.01, unpaired Student *t*-test.

16S rRNA and metagenomics sequencing can be complementary in identifying bacterial compositional changes at different taxonomic levels (34, 35). To examine microbiome composition at a higher resolution and evaluate their functional capacity, shotgun metagenomics sequencing was performed for the fecal samples collected from the same Bp 531D- and PBS-gavaged mice (Fig. 3B). Obtained data demonstrated a slight increase in bacterial richness (Fig. 4A) and consistent separation of community clusters (Fig. 4B). 47 of 196 species-level genome bins (SGBs), particularly SGBs of Lachnospiraceae, were detected with significant changes (Fig. 4C). *Bifidobacterium pseudolongum*, *Leptogranulimonas caecicola*, and *Turicibacter* sp. 1E2, which all express bile acid-modifying enzymes (e.g., bile acid hydrolases) important for controlling host bile acid and lipid metabolism (36, 37), were the top species enriched in the Bp 531D group (Fig. 4C). In addition, *Acutalibacter muris*, which is sensitive to the levels of bile acids in culture medium (38), expanded noticeably, indicating the possible influence on bile acid and cholesterol homeostasis. By contrast, *Clostridium cocleatum,* whose abundance is decreased by a probiotic power used for the treatment of colorectal cancer (39), and *Romboutsia ilealis*, which has recently been shown to stimulate cytokine expression in the ileum of broilers (40), were the defined species decreased in the Bp 531D group.

Functional profiling of the metagenomes revealed increased abundances of pathways related to coenzyme A biosynthesis, glycolysis, and microbial fermentation to acetate and lactate, whereas heme B biosynthesis, which may contribute to oxidative stress (41–43), and toxic formaldehyde metabolism were the major pathways reduced in the Bp 531D group (Fig. 4D). The biosynthesis of sulfur amino acids and assimilatory sulfate reduction, a process of generating hydrogen sulfide (H_2_S) that can be incorporated into methionine and anti-oxidant cysteine by microorganisms (44), were also reduced by Bp 531D. Along with reduced biosynthesis of anti-oxidant polyamines (Fig. 4D), these results may be indicative of a different oxidative state in the gut.

A fundamental function of gut microbiota is to break down complex carbohydrates, generating bioactive metabolites and nutrients that critically contribute to the metabolic health of the hosts (45). To gain insight into microbial carbohydrate metabolic function, we analyzed the abundance of CAZymes and predicted their substrates in the fecal metagenomes of Bp 531D- and PBS-treated mice using dbCAN3 (46). We detected 32 differentially enriched CAZymes (9.0% of total CAZymes) across five enzyme families, particularly glycoside hydrolases (GHs) (Fig. 4E). dbCAN-PUL search against the metagenomes was performed to estimate the abundances of experimentally verified CAZyme gene clusters (CGCs) and predict glycan substrates. Compared to PBS controls, Bp 531D-conditioned microbiota had reduced genetic capacity to utilize arabinan and increased abundances of CGCs for degrading β-glucan, cellulose, trehalose, and carrageenan (Fig. 4F), documenting a modified carbohydrate metabolism of the microbiome.

### Transcriptomic programming of colonic ECs

**Fig 5:**
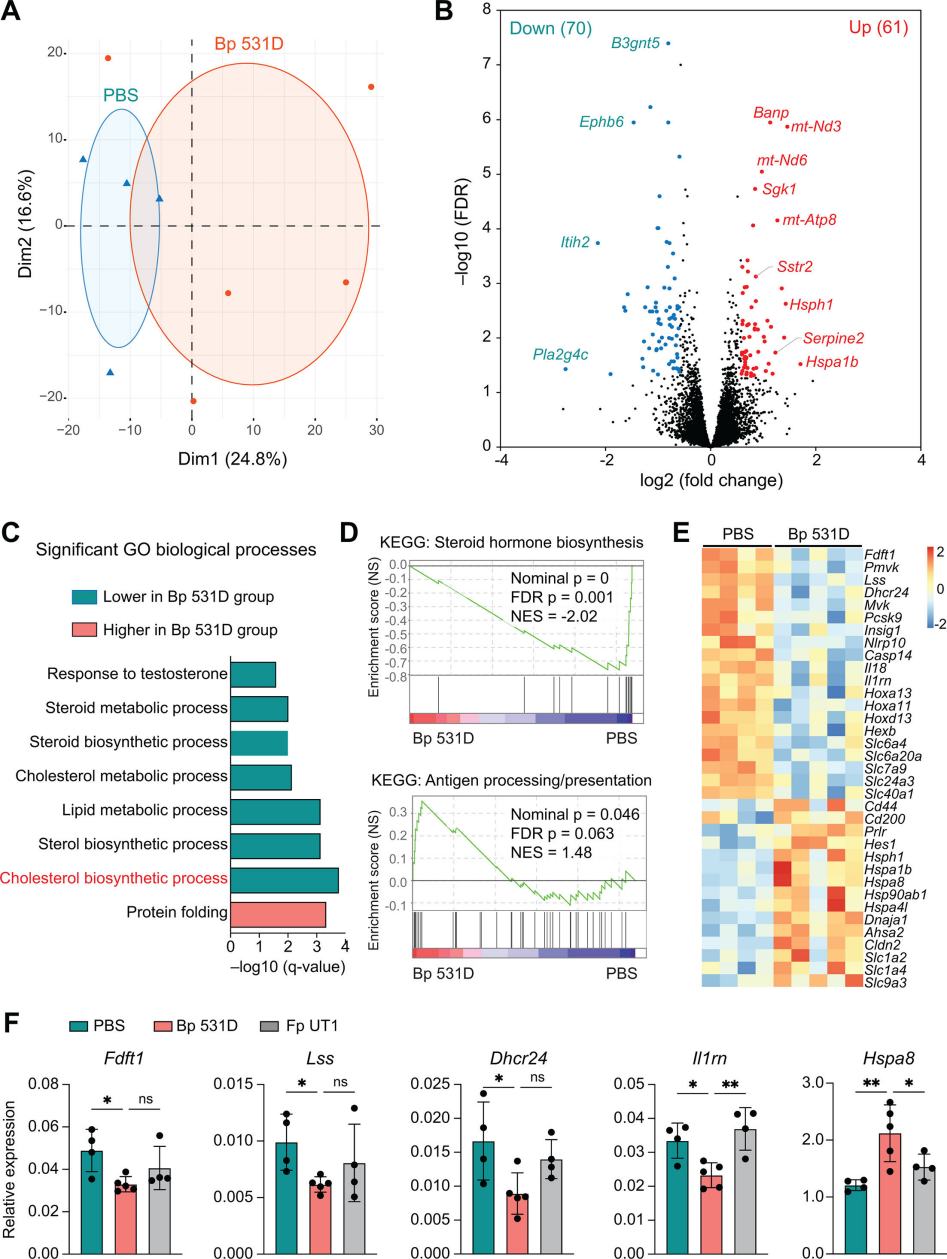
Transcriptomic programming of colonic ECs. (**A**) Principal component analysis (PCA) plot of transcriptomes of colonic ECs derived from Bp 531D- and PBS-gavaged mice (*n* = 4-5/group). (**B**) RNA-seq results depicted as a volcano plot showing differentially expressed genes (DEGs) (fold change >1.5, FDR < 0.05) between the two groups of ECs. The number of upregulated (red) and downregulated (blue) DEGs in the Bp 531D group is shown in parentheses. (**C**) Significant DAVID Gene Ontology (GO) terms related to biological process. (**D**) Representative GSEA plots showing differentially enriched KEGG pathways in colonic ECs of Bp 531D-gavaged mice versus controls. (**E**) Heatmap of DEGs. (**F**) qPCR analysis of DEG expression in the Bp 531D group versus PBS or the control bacterium group (Fp UT1). *P < 0.05 and **P < 0.01, One-way ANOVA plus Tukey’s post-test.

Ample evidence hints at the critical impacts of gut microbiota and associated metabolism on host EC signaling, leading to functional programming of the cells (47, 48). To determine whether differences in gut microbiome may be associated with alterations in EC responses, we analyzed the transcriptomes of colonic ECs isolated from mice that were gavaged with Bp 531D versus PBS for 2 weeks (Fig. 3B). The resulting two groups of transcriptomes were largely separated by principal component 1 (PC1) that explains 24.8% of the variations (Fig. 5A). Differential analysis identified a total of 131 differentially expressed genes (DEGs; fold change >1.5, false discovery rate <0.05) (Fig. 5B). The top genes upregulated by Bp 531D included mitochondrially encoded genes (e.g., *mt-Nd3*, *mt-Nd6*, and *mt-Atp8*), serum/glucocorticoid-regulated kinase 1 (*Sgk1*) that may attenuate oxidative stress by controlling mitochondrial function (49, 50), anti-apoptotic serine proteinase inhibitor member 2 (*Serpine2*) (51), and BTG3-associated nuclear protein (*Banp*), required for cell-cycle progression and cell survival (52). The top downregulated genes included protease inhibitor *Itih2*, glycosyltransferase *B3gnt5*, as well as Eph receptor 6 (*Ephb6*) and phospholipase *Pla2g4c*, found to be overexpressed in a colon cancer cell line and human colon cancer tissues (53, 54).

Probiotics such as *Lactobacillus* and *Bifidobacterium* have been shown to exhibit hypocholesterolemic effects (55, 56). The biosynthesis of cholesterols and steroids, many of which are known to influence inflammatory responses (57, 58), was the major pathway reduced in the Bp 531D group (Fig. 5C and D). Accordingly, the cholesterol-synthesizing enzymes, including farnesyl-diphosphate farnesyltransferase 1 (*Fdft1*), lanosterol synthase (*Lss*), and 24-dehydrocholesterol reductase (*Dhcr24*), had decreased expression in mice gavaged with Bp 531D, but not the control bacterium *Faecalibacterium prausnitzii* strain UT1 (Fp UT1) (Fig. 5E and F), another member of Clostridial cluster IV (59). Meanwhile, the genes encoding inflammation-associated mediators (*Nlrp10*, *Casp14*, *Il18*, and *Il1rn*), HOX transcription factors (*Hoxa11*, *Hoxa13*, and *Hoxd13*) potentially affect NF-kB signaling (60), and the β subunit of β-hexosaminidase (*Hexb*), which is activated upon inflammation (61, 62), were all transcriptionally suppressed by Bp 531D (Fig. 5E and F).

By contrast, the pathway of antigen processing and presentation (Fig. 5D) and transcripts implicated in immune activation, including *Cd44*, *Cd200*, *Prlr*, and *Hes1* (Fig. 5E), were enriched in Bp 531D mice compared to PBS controls. Heat-shock proteins can be induced by various microbial components and released by different cell types in physiological conditions to elicit an immune response and protect against oxidative stress (63, 64). Here, the transcription of a panel of heat-shock proteins (*Hsph1*, *Hspa1b*, *Hspa4l*, *Hspa8*, *Hsp90ab1*, and *Dnaja1*) and the activator of heat-shock 90 KDa protein ATPase homolog 2 (*Ahsa2*) was all stimulated by Bp 531D gavage (Fig. 5E and F), suggesting a potential role of the programmed ECs in facilitating immune activation. Claudin-2 (*Cldn2*), reported to protect mice against induced colitis (65), was also expressed at significantly higher levels in Bp 531D-gavaged mice compared to controls (Fig. 5E).

Furthermore, Bp 531D administration altered the transporting activities of the ECs, as demonstrated by elevated expression of glutamate transporter *Slc1a2*, neutral amino acid transporter *Slc1a4* (influx), and declined levels of serotonin reuptake transporter *Slc6a4*, proline transporter *Slc6a20*, and *Slc7a9* exchanging cystine (influx) for neutral amino acids (efflux) (66). Differences in ion channels, including Na^+^/H^+^ exchanger *Slc9a3*, Na^+^/Ca^2+^ exchanger *Slc24a3*, and iron exporter *Slc40a1,* were also observed (Fig. 5E), suggesting a functional programming of colonic ECs.

### Regulation of intestinal immune response at homeostasis

**Fig 6:**
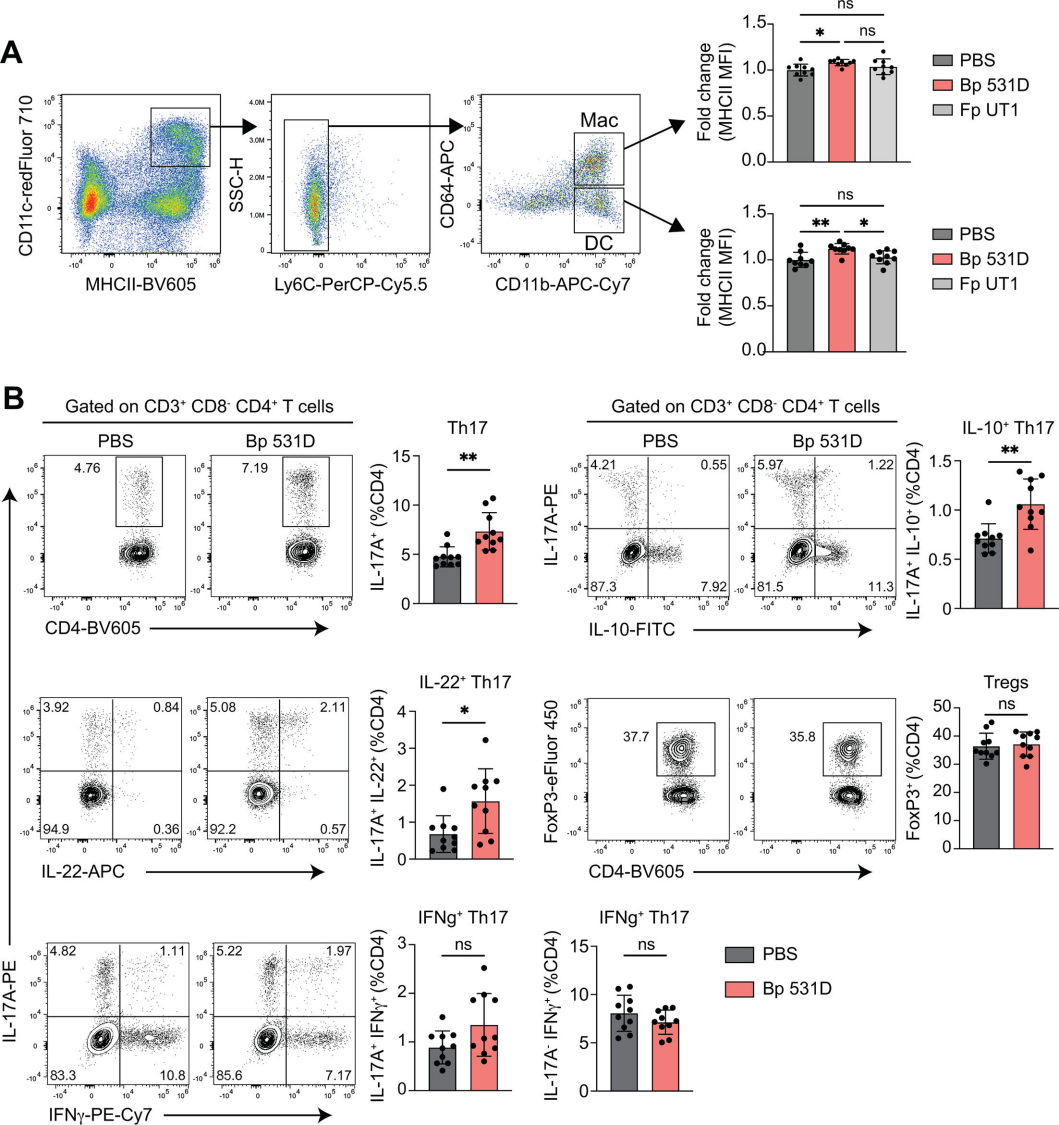
Colonic immune response in mice gavaged with Bp 531D. (**A**) Upregulation of MHC-II expression, as shown by increased mean fluorescence intensity (MFI), in colonic DCs and macrophages (Macs) isolated from mice gavaged with Bp 531D vs. PBS or Fp UT1 controls (*n* = 9/group). (**B**) Representative FACS plots and frequencies of colonic IL-17A^+^ Th17 cells (IL-10^+^, IL-22^+^, and IFNγ^+^ Th17 subsets), IL-17A^-^ IFNγ^+^ Th1 cells, and FoxP3^+^ Tregs in mice gavaged with Bp 531D or PBS for 14 days (*n* = 10/group). ns, not significant; *P < 0.05, **P < 0.01, One-way ANOVA plus Tukey’s post-test (**A**) or unpaired Student *t*-test (**B**).

Having demonstrated the profound changes in the gut microbiome and EC functions, both of which are crucial for intestinal immune regulation (47), we next examined the colonic immune responses in mice gavaged with Bp 531D or controls. The expression of MHCII was significantly increased in DCs and macrophages of mice treated with Bp 531D compared to PBS or the control bacterium (Fig. 6A), indicating the specific enhancement of cell mutation and antigen-presenting function. The colonic CD4^+^ T-cell responses were then compared between Bp 531D- and PBS-gavaged mice. Here, the generation of Th17 cells, including IL-10^+^ Th17 cells critical for anti-inflammatory function (67) and IL-22^+^ Th17 cells essential for mucosal homeostasis (68), was augmented by Bp 531D gavage, while the percentage of IFNγ^+^ Th17 cells remained unchanged (Fig. 6B). No differences in FoxP3^+^ Tregs and IL-17A^-^/IFNγ^+^ Th1 cells were observed (Fig. 6B). These results indicate that Bp 531D promotes the homeostatic immune activation in the colon.

## DISCUSSION

The human gut microbiota hosts a largely untapped wealth of immunoregulatory activities, and mining the microbiota for effector strains is, however, hampered by a lack of ability to isolate and culture individual anaerobic microbes and validate their intestinal functions using experimental animal models (5, 69). Thus far, few bacterial species have been well-elucidated for their immunological functions in mice, including segmented filamentous bacteria (SFB), which elicit a robust intestinal Th17 cell response (70), and a range of microbes, particularly *Clostridium*, which induce specific cell subsets of FoxP3^+^ Tregs (9, 12, 71, 72). While our understanding of these commensal microbes has increased tremendously over the past years, more studies are still needed to characterize novel strains that may exhibit unique intestinal functions, to mechanistically dissect how specific microorganisms may impact host physiology and how we might take advantage of the knowledge obtained to develop new probiotic treatments.

*B. porcorum* is a butyrate-producing obligate anaerobe that remains to be fully characterized. The human isolate Bp 531D differs from swine strain Bp BB10 in that it has enhanced genetic capability of folate biosynthesis and subsequent one-carbon metabolism, which is in accordance with elevated absorption of dietary folate in human infants compared to piglets (73), potentially reflecting evolutionary adaptation to specific metabolic needs of the hosts (74). While several sequencing analyses reveal its presence in animals (25–27), the role of this bacterium, as part of human gut microflora, has yet to be investigated. There is only one study reporting *B. porcorum* in the fecal metagenomes of a Chinese cohort (28). The low occurrence of the species may be due to its low abundance to be detected in microbiome sequencing analyses and/or its preferential colonization in specific human populations, as gut microbiome often varies according to age, lifestyle, disease, and geography (75, 76).

Increasing evidence suggests the co-existence of *Bifidobacterium* species and butyrate-producing bacteria in human colons, and co-incident decreases in the bacterial numbers have been reported in patients with various disorders (77). *B. porcorum* also expands after supplementation of *Bifidobacterium* in a human interventional trial (28), and its abundance, along with *Lactobacillus*, declines in the chicken microbiota after pathogen infection (26). Consistently, oral administration of Bp 531D also led to the enrichment of *Bifidobacterium* and *Lactobacillus* in mice. Although Bp 531D produces butyrate in the culture medium, its oral gavage significantly depleted the genetically closely related Clostridiaceae that also produce butyrate, which may be attributable to the bacterial competition for similar substrates (e.g., β-glucan) (78), resulting in an overall reduction of cecal butyrate in the treated mice. Whether the butyrate-producing capability of Bp 531D contributes to the bacterial fitness in the gut remains unclear, and more studies are needed to further elucidate its bifidogenic mechanisms.

Intestinal ECs critically sense luminal and microbial signaling and program their cellular molecular machinery adapting to the dynamic gut microenvironments. Alterations in gut microbiome composition and carbohydrate metabolic potential may result in differential production of metabolites that may be accessible to colonic ECs, resulting in the functional programming of the cells (47, 79). We observed that enriched metagenomic pathways of glycolysis and pyruvate fermentation, which may produce metabolites (e.g., acetate and lactate) activating mitochondrial respiration (80, 81), correlated with enhanced expression of mitochondrial DNA-encoded respiratory genes (e.g., *mt-Nd3* and *mt-Nd6*) in the ECs of Bp 531D-gavaged mice, perhaps indicating a microbial control of epithelial mitochondrial function. Elegant studies have shown that ECs can sample luminal microbial antigens to drive a regulatory Th17 cell response (82). Here, the elevated pathway of antigen processing and presentation in ECs was associated with an induction of IL-10-producing Th17 cells. In addition, DCs were also activated with the expression of MHC-II crucial for microbial-specific Th17 cell generation (83), highlighting the microbiome-dependent regulation of Th17 cells.

Cholesterol is the precursor for the biosynthesis of steroid hormones and bile acids, and a higher level of blood cholesterols (hypercholesterolemia) is one of the leading causes of atherosclerosis and cardiovascular diseases (84). Probiotic bacteria such as *Lactobacillus* and *Bifidobacterium* have been reported to exhibit cholesterol-lowering effects potentially via expressing bile acid-hydrolyzing enzymes, entrapping cholesterols, and producing functional metabolites (55, 56, 85). A recent study also shows that colonizing mice with a *Turicibacter* strain decreases the levels of serum cholesterol and modifies the host lipid profile, and the effect is linked to the bacterial capability to deconjugate bile acids (37). These bacteria (*Bifidobacterium*, *Lactobacillus*, and *Turicibacter*) were enriched in the gut microbiome of Bp 531D-treated mice, which also had increased abundances of CAZyme clusters to degrade β-glucan, a soluble fiber approved by the Food and Drug Administration for its cholesterol-lowering properties (86). Correlatively, cholesterol biosynthesis, as the major pathway, was suppressed in colonic ECs of the treated mice, providing a possible intestinal mechanism for regulating cholesterol homeostasis.

While calibrating the microbial carbohydrate metabolic function, administration of Bp 531D affected the stress responses of gut microbiome and colonic ECs: (1) the oxidant-sensitive heme B biosynthesis was the top function reduced in the Bp 531D group (2); the biosynthesis of anti-oxidant cysteine and polyamines was reduced; and (3) cell stress-associated heat-shock proteins were induced in colonic ECs. Studies have shown that controlled cellular stress induces the differentiation of Th17 cells even in the absence of TGFβ signaling (87). However, it is also reported that oxidative stress inhibits the expression of IL-17A and RORγt, the transcription factor required for Th17 cell generation, from *in vitro* differentiated murine Th17 cells (88). We showed that a properly controlled stress response was associated with the induction of a regulatory immune response, as demonstrated by increased frequencies of Th17 cells that produce IL-10 and IL-22, and the inhibition of epithelial inflammatory signaling, particularly the inflammasome-associated genes *Nlrp10* and *Il18*. These observations favor the notion that stress-associated Th17 cell response may be dependent on synergistic coordination between gut microbiome, ECs, and the immune system. In summary, our results identify a novel probiotic bacterial species with critical intestinal regulatory functions, which may be collectively considered for the design of microbiota-targeted therapeutics.

## MATERIALS AND METHODS

### Bacterial isolation and growth

Stool samples were obtained from a de-identified healthy human donor (female) with informed consent. Fecal supernatants were serially diluted and plated on reinforced clostridial agar plates containing 10 g peptone, 10 g beef extract, 3 g yeast extract, 5 g dextrose, 1 g soluble starch, 5 g sodium chloride, 3 g sodium acetate, 0.5 g cysteine hydrochloride, 0.5 mg resazurin, and 15 g agar per liter (pH 6.8). After ~48 hours of incubation, single colonies were grown overnight in reinforced clostridial broth (RCB), followed by restreaking onto a new RCB agar plate. Single colonies were picked from the second agar plate and cultured in RCB for genomic DNA isolation. 16S rRNA genes were amplified from these colonies using universal 16S primers (Table S1), purified, and sequenced for bacterial species identification. Bacteria were grown at 37°C and all the procedures were conducted in an anaerobic chamber (model AS-500, Anaerobe Systems) with a gas mixture of 90% nitrogen, 5% carbon dioxide, and 5% hydrogen.

### Whole-genome sequencing

The complete genome of Bp 531D was obtained using the complementary Oxford Nanopore and Illumina sequencing. Briefly, bacterial genomic DNA (gDNA) was extracted and concentrated using a Quick-DNA Miniprep kit (Zymo Research) and a DNeasy PowerClean Cleanup kit (Qiagen), respectively. gDNA libraries were constructed using a Native Barcoding kit to be sequenced on a MinION system (Oxford Nanopore Technologies) and an Illumina DNA Prep kit to be sequenced on the Illumina NovaSeq X plus platform. Sequencing was performed at the University of Florida NexGen DNA Sequencing Core Facility (ICBR; RRID:SCR_019152). Nanopore reads (13.3 Gb) were filtered using the Filtlong tool (v0.2.1) to keep high-quality long reads only. The Filtlong parameters were minimum length 2,000, keep percent 90, and target bases 8,000,000,000. Parameters were set up to keep long reads. Filtered long nanopore reads (read length N50 = 7.4 kb, N90 = 4.7 kb) were used for *de novo* assembly using Flye (v2.9.5), resulting in a single circular contig (2,987,257 bp; 2,748× coverage). Illumina reads (2 × 150 bp) were trimmed and filtered to remove low-quality bases and reads using Trim_galore (v0.6.6). The *de novo* assembled draft genome was polished with 35.4 million Illumina reads using pilon (v1.23). Obtained high-quality complete genome (2,990,647 bp) was annotated, and the phylogenetic tree was built using the Bacterial and Viral Bioinformatics Resource Center (BV-BRC). The prophage and ICEs were predicted using PHASTEST and ICEfinder, respectively. The detection of transposases was carried out using Prokka, while transposons were predicted via the Tncomp_finder tool and the TnCentral database. The CAZymes, CGCs, and their substrates were predicted using dbCAN3 (46).

### Mice

C57BL/6 mice were obtained from Jackson Laboratory and maintained under specific pathogen-free conditions. Age-matched (8–10 weeks old) and sex-matched mice were used for experiments. Female mice were mixed and randomly separated into groups before gavage.

Bp 531D glycerol stocks were prepared as previously described (59). Briefly, single colonies were grown in 500 mL of RCB for ~24 hours. Cells were harvested by centrifugation, resuspended in anaerobic PBS containing 25% glycerol, aliquoted, and stored at −80°C. For gavaging, bacterial stocks diluted in PBS-glycerol solution or the control PBS-glycerol were transferred to a 1 mL syringe pre-mounted with an animal feeding needle (Fine Science Tools), and then kept in an airtight AnaeroPouch Bag (Mitsubishi) until animal feeding. Mice were gavaged every 2 days with Bp 531D (10^9^ CFU/mouse), Fp UT1 (10^9^ CFU/mouse), or PBS, and sacrificed 2 days after the last gavage (day 14) to collect colon tissues and fecal samples. For examining bacterial colonization, feces were collected on day 0 (baseline), and days 1, 2, and 3 after a single gavage or 14 days after 7 gavages to quantify Bp 531D-specific 16S rRNA copies based on the absolute quantification method using a 16S rRNA standard curve. Quantitative real-time PCR (qPCR) was performed on a QuantStudio 6 Pro real-time PCR system (Thermo Fisher) using the PowerUp SYBR Green Master Mix (Thermo Fisher).

### High-performance liquid chromatography (HPLC)

The levels of acetate and butyrate in the culture supernatants were quantified using HPLC essentially as previously described (59). SCFAs were extracted from the cecum contents of Bp 531D- and PBS-gavaged mice (*n* = 9-10/group) as described previously (89) with modifications. Briefly, cecal samples were weighted and homogenized in 1 mL of sterile water, followed by the addition of 400 μL of concentrated HCl to preserve the volatile SCFAs. After vigorous vortexing, 5 mL of methylene chloride (Sigma) was mixed with the samples at room temperature for 20 min. Samples were then centrifuged at 1,500 *× g* for 5 min, and the bottom organic phase (3-4 mL) was transferred to a new tube in which 400 μL of 1 N NaOH was added. After mixing at room temperature for 20 min and subsequent centrifugation at 1,500 *× g* for 5 min, the top aqueous phase (~350 μL) was collected and mixed with 10% HCl (~35 μL). For standard curves, acetate, propionate, and butyrate were mixed in equal molar concentrations and serially diluted (40, 20, 10, 5, 2.5, and 1.25 mM). These standards were also subjected to methylene chloride extraction as described above to normalize the extraction efficiency. Cecal extracts and standards were passed through a 0.22 μm filter (EMD Millipore) before loading to the Agilent 1220 Infinity II LC system. Separations were performed using 4 mM H_2_SO_4_ as the mobile phase in a fermentation monitoring column (7.8 × 150 mm, Bio-Rad), and signals were detected using a variable wavelength detector with a wavelength set at 210 nm. Quantification was based on the peak area and the standard curve of SCFAs. Data were normalized to the weights of the cecum contents.

### Gut microbiome analysis

Fresh feces (2–3 pellets) were collected from mice gavaged with Bp 531D or PBS for 14 days (*n* = 4/group). The fecal microbiome was profiled by both 16S rRNA and shotgun metagenomic sequencing. For 16S rRNA analysis, libraries were constructed (48, 90) and sequenced to a depth of >83,459 paired-end reads (2 × 300 bp) on an Illumina Miseq instrument. Data were processed and analyzed using QIIME2 (v2022.8). For metagenomic sequencing, DNA libraries were constructed using an Illumina DNA Pre kit, and 50-73 million paired-end Illumina reads (2 × 150 bp) were obtained. After removing low-quality sequences and reads mapping to the mouse genome (GRCm38), cleaned metagenomics reads were subjected to taxonomic profiling using the MetaPhlAn4 pipeline (v4.1.1) against the MetaPhlAn database (mpa_vJun23) and the ChocoPhlAn pan-genome database (mpa_vJun23_CHOCOPhlAnSGB_202307). The unclassified SBG taxa were further annotated to the Genome Taxonomy Database (GTDB) using mpa_vJun23_CHOCOPhlAnSGB_202307_SGB2GTDB.tsv from the MetaPhlAn4 pipeline. Linear discriminant analysis effect size (LEfSe) was performed to identify bacterial taxa that significantly (LDA score >2) contributed to compositional differences at the species level. Functional analysis of the fecal metagenomes was performed using HUMAnN3 (91). CAZyme mapping and substrate prediction were conducted essentially as described previously (59).

### RNA-seq

Colonic ECs were isolated from an independent cohort of mice gavaged with Bp 531D or PBS for 14 days (*n* = 4-5/group) as previously described (48, 92). CD45^−^ CD31^−^ TER-119^−^ EpCAM^+^ ECs were FACS-sorted using a BD FACSAria Fusion flow cytometer. Total RNA was isolated using an RNeasy Plus Micro Kit (Qiagen), and RNA libraries were constructed and sequenced as described (93). Obtained raw reads (36.2 million on average; 2 × 150 bp) were aligned to the mouse reference genomes (GRCm38) using STAR v2.7.5c. Normalized transcripts per million (TPM) were generated using RSEM v1.3.3. DESeq2 was used to determine significantly expressed genes based on the criteria (TPM >1, false discovery rate <0.05, fold-change >1.5). Gene set enrichment analysis (GSEA) was performed using DAVID (https://david.ncifcrf.gov/). Representative GSEA plots were generated using GSEA v4.1.0 based on 1,000 permutations. For DEG validation, cDNA was generated using the colonic ECs isolated from mice gavaged with Bp 531D, Fp UT1, or PBS. Fp UT1 was chosen as a control bacterium as it is also a member of Clostridial cluster IV. The relative quantification (2*^–ΔCt^*) was used in qPCR to determine the expression level of the target genes normalized to *Gapdh*. Sequences of primers can be found in Table S1.

### Isolation of gut immune cells and flow cytometry

Cell isolation was performed as described previously (93). DCs and macrophages were isolated without Percoll density gradient centrifugation (94). CD4^+^ T cells were analyzed for two cohorts of mice gavaged with Bp 531D or PBS for 14 days (*n* = 10/group). Independent cohorts of mice were used to profile DCs and macrophages by including Fp UT1 as a control bacterium (*n* = 9/group). Mouse FcR Blocking Reagent was used to block non-specific binding to Fc receptors before surface staining. For cytokine staining, cells were stimulated with 50 ng/mL PMA and 500 ng/mL ionomycin for 4 hours and Brefeldin A (2 μg/mL) for 2 hours. The live and dead cells were discriminated by Live/Dead Fixable Aqua or Violet Dead Cell Stain Kit (Thermo Fisher). Antibodies were purchased from Thermo Fisher, BioLegend, or Cytek. Sample acquisition was performed using the Cytek Northern Lights flow cytometer and analyzed using FlowJo software (v10.10). Gating strategies for immune populations are shown in Fig. S3.

### Statistical analysis

Statistics were performed using unpaired Student’s *t*-test (two groups) or one-way ANOVA followed by Tukey’s post-test (three groups) implemented in GraphPad Prism v10.2.3. *P* < 0.05 was considered significant: *P < 0.05; **P < 0.01; ***P < 0.001; ns, not significant.

## Data Availability

All data associated with this study are present in the paper and the supplemental material. The metagenomic and transcriptomic raw FastQ files have been made publicly available under the NCBI BioProject accession numbers PRJNA1209382 and PRJNA1209383, respectively. The complete genome sequence of Bp 531D is available under accession number CP178353.1.

## SUPPLEMENTAL MATERIAL

Supplemental material msystems.00790-25-s0001.pdf Fig. S1 to S3 and Table S1.

ASM does not own the copyrights to Supplemental Material that may be linked to, or accessed through, an article. The authors have granted ASM a non-exclusive, world-wide license to publish the Supplemental Material files. Please contact the corresponding author directly for reuse.
