# Supplementary material for: Genomic and functional characterization of a Butyricicoccus porcorum strain isolated from human gut microbiota
Source: mSystems. 2025 Jul 9;10(8):e00790-25. doi: 10.1128/msystems.00790-25 (PMC12363227; doi:10.1128/msystems.00790-25)
Supplement: Supplemental material — Fig. S1 to S3 and Table S1. [file msystems.00790-25-s0001.pdf]

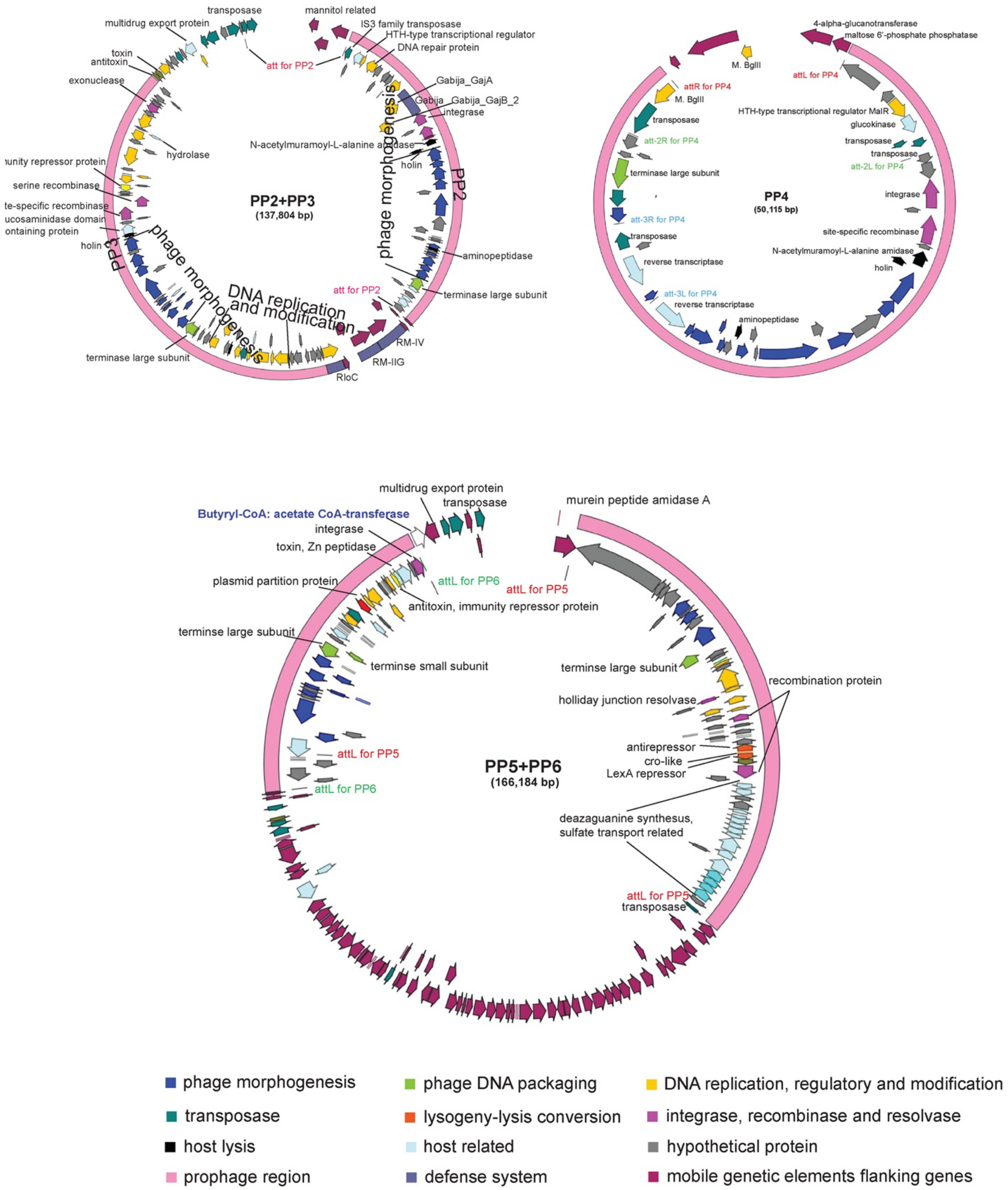

FIG S1 Genomic diagrams of prophage regions in Bp 531D genome. Genomic annotation of prophages (PPs) 2 and 3, prophage 4, prophages 5 and 6 shown as three circular maps. Genes with different function are in different colors. Attachment (*att*) sites are indicated. These prophages carry phage replication, DNA packaging, morphogenesis, lysogeny and lysis conversion and host lysis related genes, as well as host immunity related genes such as defense system, toxin-antitoxin system, and multidrug resistant genes. The prophage regions harbor or are flanked by various transposases, implying its potential active and super dynamic genomic state.

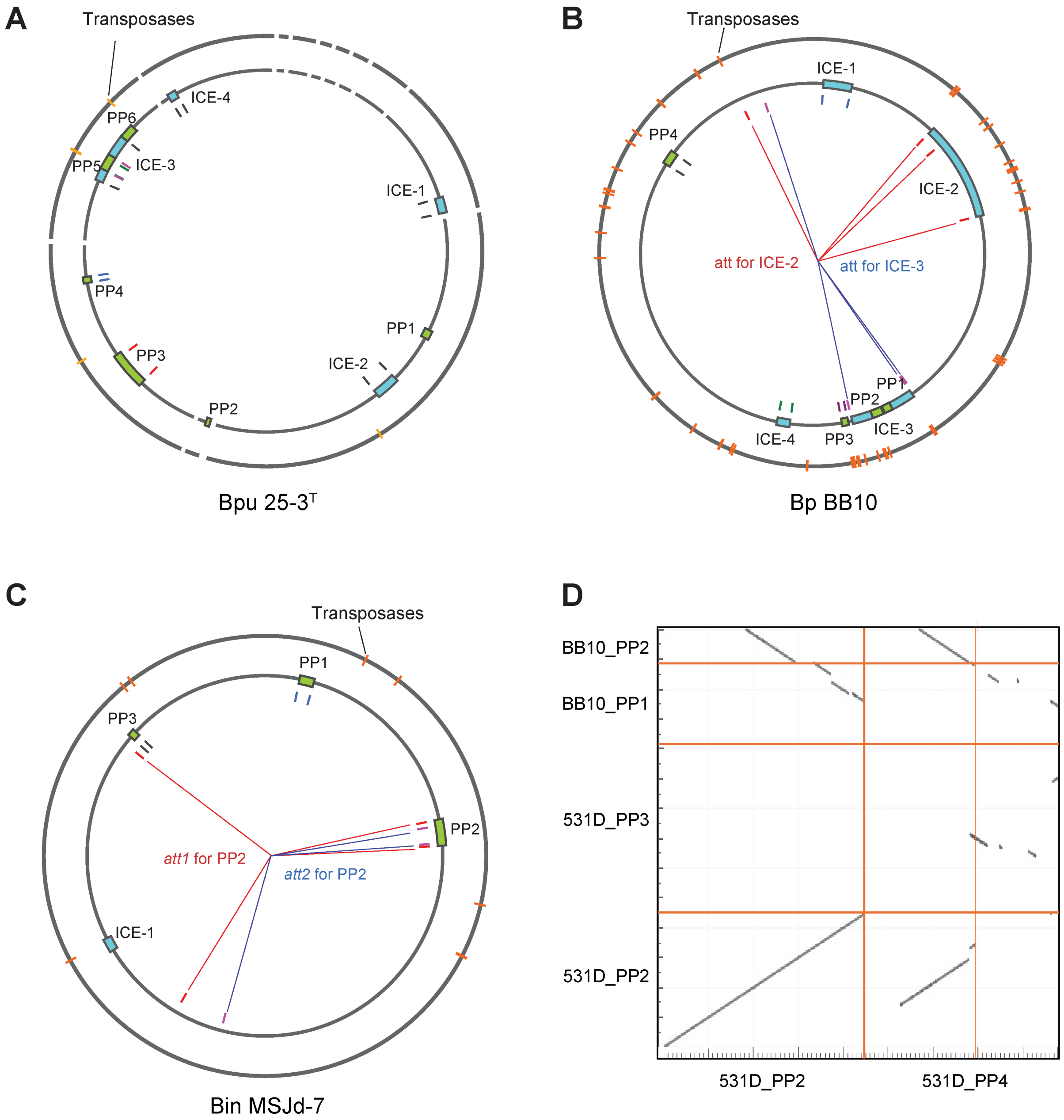

FIG S2 Distribution of mobile genetic elements (MGEs) in selected *Butyricicoccus* genomes. Prophages (PP), integrative conjugative elements (ICEs), and transposases are shown in green, blue, and orange, respectively **(A-C)**. Attachment sites are also indicated with different colors. **(D)** Comparative analysis of prophages showing the regions of similarity, inversions, repeats, or rearrangements. The two prophages of Bp 531D (531D-PP2 and 531D-PP4) exhibit a high coverage and similarity with BB10-PP1 and BB10-PP2 found in Bp BB10. 531D-PP4 shows partial overlap with both 531D-PP2 and 531D-PP3.

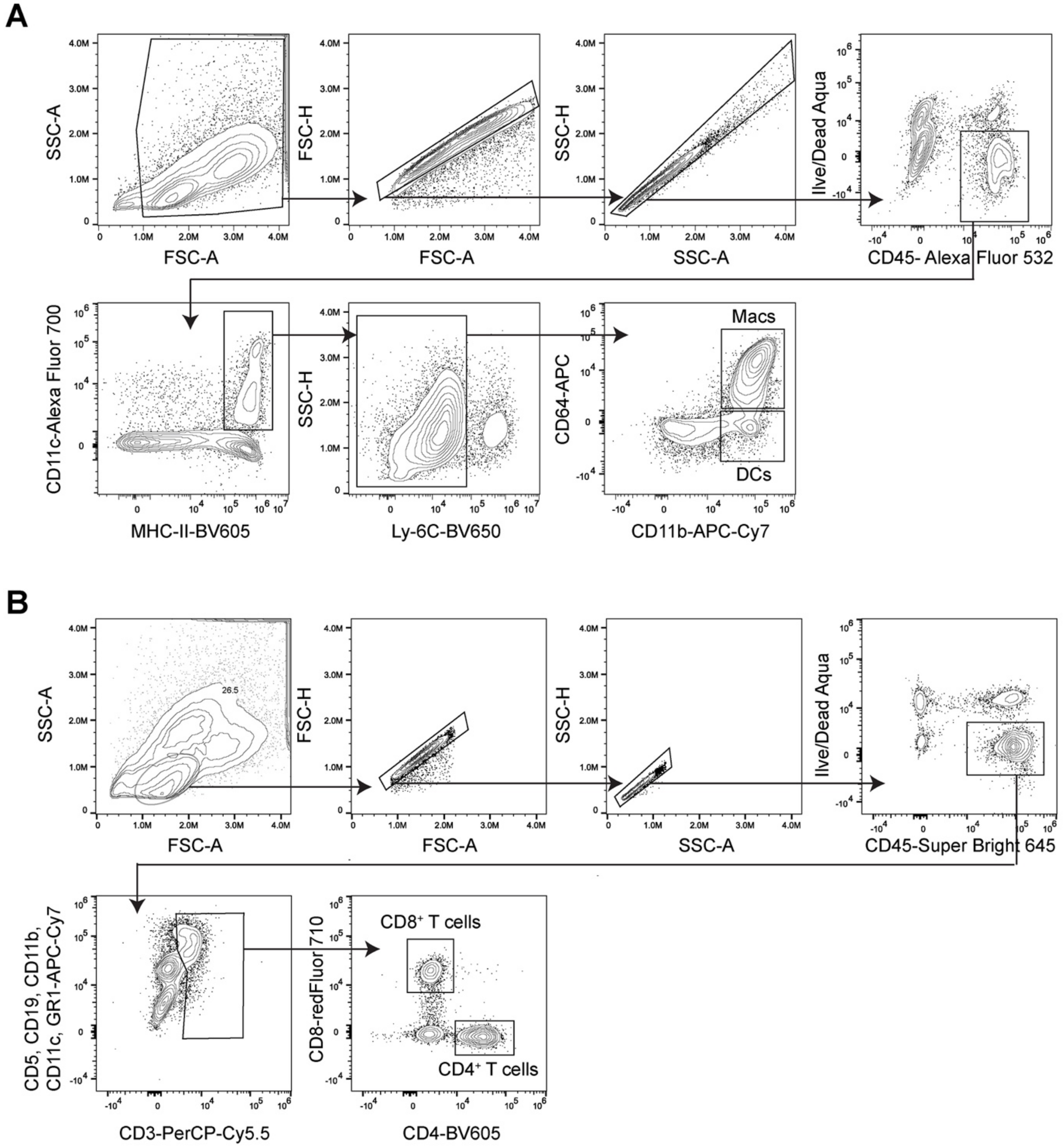

FIG S3 Gating strategies for colonic immune cell populations. **(A)** Flow gating strategies for colonic dendritic cells (DCs) and macrophages (Macs). After exclusion of doublets and dead cells. DCs were defined as CD45$^+$ CD11C$^+$ MHC-II$^+$ Ly-6C$^-$ CD11b$^+$ CD64$^-$ cell populations, and Macs were CD45$^+$ CD11C$^+$ MHC-II$^+$ Ly-6C$^-$ CD11b$^+$ CD64$^+$. **(B)** Flow gating strategies for colonic T cells, which were CD45$^+$ CD3$^+$ CD8$^-$ CD4$^+$.

Table S1. Primers used for qPCR in this study

Gene	Forward (5' to 3')	Reverse (5' to 3')
Universal 16S	AGAGTTTGATCMTGGCTCAG	GGTTACCTTGTTACGACTT
Bp 531D 16S	TGGGTGTAAAGGGCGAGTAG	CCGGGGTTAAGCCCCGAC
Gapdh	GGTGAAGGTCGGTGTGAACG	CTCGCTCCTGGAAGATGGTG
Hspa8	TCTCGGCACCACCTACTCC	CTACGCCCGATCAGACGTTT
Il1rn	GCTCATTGCTGGGTACTTACAA	CCAGACTTGGCACAAGACAGG
Dhcr24	CTCTGGGTGCGAGTGAAGG	TTCCCGGACCTGTTTCTGGAT
Fdft1	ATGGAGTTCGTCAAGTGTCTAGG	CGTGCCGTATGTCCCCATC
Lss	TCGTGGGGGACCCTATAAAAC	CGTCCTCCGCTTGATAATAAGTC
